# The Causes of Cough in Children

**Published:** 1899-08-26

**Authors:** 


### THE CAUSES OF COUGH IN CHILDREN.

From an analysis of 700 consecutive cases of cough in children between six months and twelve years of age, Dr. Porter Parkinson concludes that this is a symptom which is associated with diseases of the throat more often than with any other condition. Out of the 700 cases noted it was due to acute or chronic enlargement of the tonsils in 170 cases; to varying degrees of bronchitis in 143 cases; to constipation in 69 cases; to gastro-enteritis in 53 cases; to adenoids, well marked, in 58 cases; to pharyngitis in 51 cases; and to other diseases in various smaller numbers, Taking them altogether, they were due to chest diseases in 41 per cent. of the cases, to throat diseases in 40 per cent., to gastro-intestinal disease in 23 per cent., and to other causes in 6 per cent. It is to be noted that ear troubles are not mentioned as a cause of cough, the fact being that in the cases in which the ear was found diseased there was always some morbid state of the throat present which seemed in itself quite competent to produce cough. Among other things pointed out in this very practical paper is the fact that bronchiectasis and enlargement of the mediastinal glands may in some cases cause a cough which may exactly simulate that of whooping-cough. Another condition which may cause a cough identical with whooping-cough is enlargement of the bronchial glands, generally of tuberculous origin. A class of cases which it is especially necessary to beware of is that in which the cough is but a symptom of gastro-intestinal disease. These cases amount to 23 per cent of the whole. The conditions present in them were constipation, vomiting and diarrhœa, and intestinal worms, and in many of them the cough was so prominent a symptom that, before the cases came to the hospital, treatment had been specially directed to it without result. It is worth observing that in some of the throat cases, due to enlarged tonsils, sometimes complicated with adenoids, an occasional rhonchus may be present in the lungs, a fact which may lead to erroneous diagnosis, but treatment directed to the lungs will not effect a cure if the tonsillar affection be disregarded.

